# Characterization and In Vitro Probiotic Assessment of *Alkalihalobacillus wakoensis* as New Potent Probiotic Candidate

**DOI:** 10.1002/fsn3.71031

**Published:** 2025-09-26

**Authors:** Ali Zarei, Ahmad Gholami, Zahra Kanannejad, Aydin Berenjian, Seyed Hesamodin Nabavizadeh, Milad Mohkam

**Affiliations:** ^1^ Department of Physiology, Estahban School of Paramedical Sciences, School of Nursing Hazrat Zahra (P.B.U.H) Abadeh, Shiraz University of Medical Sciences Shiraz Iran; ^2^ Biotechnology Research Center Shiraz University of Medical Sciences Shiraz Iran; ^3^ Department of Pharmaceutical Biotechnology, School of Pharmacy Shiraz University of Medical Sciences Shiraz Iran; ^4^ Allergy Research Center Shiraz University of Medical Sciences Shiraz Iran; ^5^ Department of Chemical and Biological Engineering Colorado State University Fort Collins Colorado USA; ^6^ Department of Agricultural and Biological Engineering Pennsylvania State University University Park Pennsylvania USA; ^7^ Department of Allergy and Clinical Immunology, Namazi Hospital Shiraz University of Medical Sciences Shiraz Iran

**Keywords:** *Alkalihalobacillus wakoensis*, alkaliphilic *Bacillus*, *Bacillus clausii*, probiotics

## Abstract

*Bacillus* spp. probiotics have been shown to be promising due to their resilience in the gut. This study evaluated the in vitro probiotic potential of *Alkalihalobacillus wakoensis* PTCC 1596, using *Bacillus clausii* ATCC 700160 as a control. In vitro probiotic characteristic assays were conducted to compare *A. wakoensis* and *B. clausii* (control) in terms of acid and bile tolerance, enzymatic activity, antimicrobial potential, antibiotic susceptibility, biofilm formation, hydrophobicity, autoaggregation, enterotoxin gene presence, and cytotoxicity. *A. wakoensis* spores demonstrated high survival (95.6% ± 0.9%) at pH 2.5 for 3 h and tolerance to 0.5% oxgall bile salts. The strain produced catalase, protease, and nattokinase, and exhibited moderate cell surface hydrophobicity (47.1% ± 3.12% to xylene) and autoaggregation (7.0% ± 1.2% after 1 h). It showed sensitivity to several antibiotics (e.g., chloramphenicol, gentamicin) and lacked detectable plasmids and tested enterotoxin genes (*hblA*/*hblC*/*hblD*, *nheA*/*nheB*, *bceT*, and *cytK*). Cytotoxicity assays using HepG‐2 cells indicated non‐toxicity, with supernatants potentially enhancing cell viability. While exhibiting lower biofilm formation than *B. clausii* , *A. wakoensis* displayed promising traits. These findings suggest *A. wakoensis* PTCC 1596 is a potential probiotic candidate warranting further investigation, particularly given its alkaliphilic nature.

## Graphical abstract

*Alkalihalobacillus wakoensis* ATCC 21832 demonstrated excellent acid (95.6% survival at pH 2.5) and bile tolerance, produced catalase, protease, and nattokinase, and exhibited broad antimicrobial activity. The strain was sensitive to several antibiotics, formed moderate biofilm, showed high hydrophobicity, adhered to epithelial cells, and enhanced HepG‐2 viability without toxin genes or hemolysis. These results highlight its potential as a safe, alkaliphilic probiotic candidate with multifunctional health‐promoting properties.

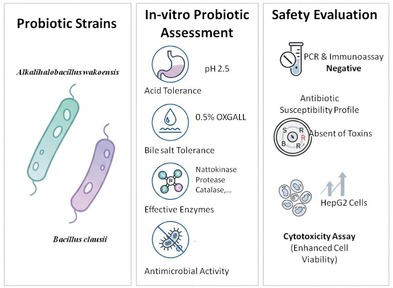

## 1 Introduction

The genus *Bacillus* belongs to a broad group of Gram‐positive, rod‐shaped, spore‐forming bacteria that are found universally in nature and are being used for their applications in biotechnology, agriculture, and human health (Lu et al. 2024; Payne et al. 2024). Among the many applications, *Bacillus* species have emerged as promising probiotic candidates due to their environmental resilience, spore‐forming ability, and, importantly, the intrinsic resistance of their spores to extreme conditions, including those found in the gut (GIT) (Payne et al. 2024). The robustness of *Bacillus* spores allows them to withstand harsh conditions such as heat, desiccation, and acidic environments. Unlike conventional lactic acid bacteria like *Lactobacillus* and *Bifidobacterium*, which require cold storage and controlled conditions, *Bacillus* probiotics, in their spore form, can survive and function under a wider range of conditions. Probiotics typically comprise beneficial bacteria from genera such as *Lactobacillus* (e.g., *L. acidophilus* , *L. rhamnosus* ), *Bifidobacterium* (e.g., *B. longum* , *B. bifidum* ), and spore‐forming *Bacillus* species (e.g., *B. subtilis* , *B. coagulans* ), along with non‐bacterial options like the yeast *Saccharomyces boulardii* (FAO/WHO 2002; Hill et al. 2014). These microorganisms are selected for their ability to confer health benefits, including gut microbiota modulation, pathogen inhibition, and immune support when consumed in sufficient quantities. This makes them ideal for probiotic formulations that need to be stable during storage, processing, and transit through the acidic environment of the stomach (Payne et al. 2024).

*Bacillus* spp., including *Shouchella clausii* (formerly known as *Bacillus clausii* ), is marketed as Enterogermina and is a widely used *Bacillus* species in the probiotic industry. *S. clausii* is known for its intrinsic antibiotic resistance, high survival in the gastrointestinal tract (GIT), and alkalophilic nature, which enables it to thrive in specific environmental habitats. These characteristics make it a robust candidate for probiotic applications (Payne et al. 2024; Ghelardi et al. 2015). Clinical studies have shown that *S. clausii* (Enterogermina) has immunomodulatory effects and can restore gut flora balance, making it a suitable option for individuals experiencing dysbiosis due to antibiotic use or other factors. However, despite its efficacy and safety profile, *S. clausii* represents only a small fraction of the *Bacillus* genus that could be used for probiotic applications (Ghelardi et al. 2015; Sadrimovahed and Ulusoy 2024). Therefore, exploring other *Bacillus* species with unique physiological traits could uncover alternative or complementary benefits and broaden the applications of *Bacillus*‐based probiotics in health and disease.

*Bacillus wakoensis* , recently reclassified as *Alkalihalobacillus wakoensis*, is an alkaliphilic, spore‐forming bacterium isolated from high pH environments, such as soda lakes and alkaline springs (Grant et al. 1990; Joshi et al. 2022). Alkaliphilic *Bacillus* strains, which thrive in high pH conditions, may offer specific benefits in the gastrointestinal tract (GIT), particularly in the small intestine where the pH is naturally higher (Grant et al. 1990; Bernardeau et al. 2017). The unique alkaliphilic properties of *B. wakoensis* could enable it to function effectively in environments where conventional probiotics may lose viability, thus broadening the application of *Bacillus* probiotics to populations with altered GIT pH due to dietary or health‐related factors (Elshaghabee et al. 2017). Moreover, its resilience to various stressors suggests potential as a dietary supplement, especially where traditional probiotics may be ineffective (Payne et al. 2024). Despite its unique characteristics, there has been limited research on *A. wakoensis* as a probiotic, making this study one of the first to comprehensively assess its probiotic potential.

This study aims to evaluate the in vitro probiotic properties of *A. wakoensis* PTCC 1596, using *S. clausii* ATCC 700160 as a control. By comparing attributes in terms of antimicrobial activity, biofilm formation, autoaggregation, hydrophobicity, enzymatic profile, antibiotic susceptibility, and safety factors like cytotoxicity and absence of toxins, this study seeks to position *A. wakoensis* relative to an established probiotic benchmark (Elshaghabee et al. 2017; Dabiré et al. 2022). These characteristics are crucial for determining probiotic suitability as they relate to gut colonization, pathogen inhibition, safe host interaction, and resilience under environmental pressures (Elshaghabee et al. 2017; Mohkam et al. 2020). The findings contribute to our understanding of alkaliphilic *Bacillus* strains while highlighting their potential for targeted dietary use.

To the best of our knowledge, this study provides one of the first comprehensive assessments of *A. wakoensis* as a potential probiotic, focusing on its unique alkaliphilic properties and comparing its probiotic attributes to those of established strains like *S. clausii* ATCC 700160. While previous studies have explored certain characteristics of *A. wakoensis*, this work offers a broader evaluation of its probiotic potential, safety, and suitability for dietary applications. By comparing *A. wakoensis* with the well‐established *S. clausii* ATCC 700160, this study aims to provide a comprehensive assessment of the probiotic potential and safety of these strains by examining key attributes such as acid and bile tolerance, enzymatic activity, hydrophobicity, autoaggregation ability, biofilm formation, antibiotic susceptibility, cytotoxicity, and absence of toxins. By comparing these characteristics with those of *S. clausii* , this research seeks to position *A. wakoensis* relative to an established probiotic benchmark. These findings contribute to our understanding of alkaliphilic *Bacillus* strains while highlighting their potential for targeted dietary use. In this study, we employed a range of in vitro assays to evaluate the probiotic potential of *A. wakoensis* PTCC 1596, including acid and bile tolerance, antimicrobial activity, biofilm formation, autoaggregation, hydrophobicity, enzymatic profile, antibiotic susceptibility, cytotoxicity, and the absence of toxins. These assays were chosen based on established guidelines for probiotic characterization (FAO/WHO 2002; Hill et al. 2014) and are widely used in the field to assess the functional and safety attributes of potential probiotic strains (Elshaghabee et al. 2017; Mohkam et al. 2020). The use of *S. clausii* ATCC 700160 as a control strain provides a valuable benchmark, as it is a well‐characterized probiotic with a proven track record in clinical applications (Ghelardi et al. 2015). By comparing *A. wakoensis* to this established strain, we aim to contextualize its probiotic potential and highlight its unique attributes, particularly its alkaliphilic nature, which may offer advantages in specific gastrointestinal environments.

## 2 Materials and Methods

### 2.1 Bacterial Strains and Growth Conditions

*Alkalihalobacillus wakoensis* PTCC 1596 (ATCC 21832) and *Shouchella clausii* IBRC‐M 10589 (ATCC 700160) (used as a control) were obtained from the Iranian Research Organization for Science and Technology (IROST) and the Iranian Biological Resource Center (IBRC), respectively. Strains were maintained on Alkaline *Bacillus* medium slopes (glucose 10 g/L, peptone 5 g/L, yeast extract 5 g/L, K_2_HPO_4_ 2.5 g/L, MgSO_4_·7H_2_O 1 g/L, agar 15 g/L; pH adjusted to 9.5 ± 0.2) at 4°C. Prior to experimentation, each strain was subcultured twice in Alkaline *Bacillus* medium. For long‐term preservation, the *Bacillus* strains were inoculated in Alkaline *Bacillus* medium containing 40% (w/v) glycerol and stored at −70°C.

### 2.2 Preparation of Spore Suspension

Bacterial strains were streaked onto Alkaline *Bacillus* medium plates to promote aseptic spore formation and incubated at 30°C for 5 days. After incubation, biomass was gently scraped using sterile loops and suspended in sterile normal saline (0.9% NaCl). The suspension was heat‐treated at 80.0°C ± 0.5°C for 30 min in a calibrated water bath with continuous gentle mixing to inactivate vegetative cells while preserving spore viability. Samples were immediately cooled on ice to prevent premature germination. The suspension was centrifuged at 3000 *g* for 10 min at 4°C to pellet the spores, discarding the supernatant, and resuspending the pellet in sterile 0.9% NaCl. The suspended spores were carefully collected and diluted with sterile 0.9% NaCl to achieve a final concentration of 5.2 ± 0.5 × 10^10^ spores/mL, confirmed by the malachite green staining method and viable plate counts on Alkaline *Bacillus* agar incubated at 30°C for 48 h (Berenjian et al. 2011).

### 2.3 Acid and Bile Salt Tolerance

The acid and bile salt tolerance of bacterial spores and vegetative cells was assessed following the method of Duc et al. (2004) with modifications. Acid tolerance was evaluated by inoculating 1 mL of spore suspension (5.2 × 10^9^ spores/mL) into 9 mL of tryptic soy broth (TSB) adjusted to pH 2.5 (ad1020; ADWA, Hungary) with sterile 1 N HCl at 25°C. Samples (0.5 mL) were taken immediately (0 h) and after 3 h incubation at 37°C, neutralized with 50 μL 1 N NaOH, serially diluted in sterile 0.85% NaCl with 0.1% peptone, and plated on Alkali *Bacillus* agar for viable counts after 24 h at 30°C. Bile salt tolerance was assessed by spotting 10 μL of 48‐h‐old cultures onto alkaline TSA supplemented with 0.5% (w/v) Oxgall (bile salts) (Sigma‐Aldrich, USA) and were incubated at 30°C for 72 h. The presence of growth on alkaline TSA supplemented with 0.5% Oxgall was regarded as bile salt tolerance bacterium (Duc et al. 2004). Spores were tested for acid tolerance (using heat‐treated suspensions), while vegetative cells (from 48‐h cultures) were used for bile tolerance and other assays (e.g., antimicrobial, adhesion) to reflect GIT‐relevant states.

### 2.4 Determination of Antimicrobial Activity

The antimicrobial efficacy of *A. wakoensis* and *S. clausii* (as control) against a panel of clinically relevant bacterial strains was evaluated. Target microorganisms included *Staphylococcus aureus* ATCC 25923, *Staphylococcus epidermidis* ATCC 12228, *Pseudomonas aeruginosa* ATCC 27853, *Bacillus cereus* ATCC 11778, *Proteus vulgaris* ATCC 13315, *Escherichia coli* ATCC 25922, *Salmonella typhi* ATCC 6539, and *Shigella dysenteriae* ATCC 11835. Overnight cultures of each bacterial strain were prepared by inoculating Brain Heart Infusion (BHI) broth (HiMedia, India) and incubating at 37°C for 24 h. Subsequently, bacterial suspensions were standardized to a concentration of 1.5 × 10^5^ colony‐forming units (CFU)/mL. Aliquots of 100 μL of each standardized bacterial suspension were dispensed into 96‐well microplates. Serial two‐fold dilutions (1/2 to 1/28) of *A. wakoensis* and *S. clausii* cell‐free supernatant (CFS) (100 μL) were then added to the respective wells. Sterile BHI broth served as a negative control. Following a 24‐h incubation period at 37°C, the minimum inhibitory concentration (MIC) was determined visually as the lowest concentration of the test compound that resulted in the absence of visible bacterial growth. The CFS prepared from overnight BHI broth cultures was centrifuged at 10,000 *g* for 10 min at 4°C, filter‐sterilized (0.2 μm), and pH‐neutralized to 7.0 ± 0.2 with 1 N NaOH.

The minimum bactericidal concentration (MBC) of *A. wakoensis* and *S. clausii* was determined by subculturing 10 μL from clear wells (MIC and higher) onto TSA plates, incubated at 37°C for 24 h, with MBC defined as the lowest CFS concentration yielding no visible colony growth, corresponding to a ≥ 99.9% reduction in viable cells per Clinical and Laboratory Standards Institute (CLSI) standards. All MIC and MBC assays were conducted in triplicate (Amigh et al. 2024).

### 2.5 Antibiotic Susceptibility Examination

Antibiotic susceptibility was determined by the disk diffusion method on Mueller–Hinton agar (HiMedia, India) following CLSI (2014) standards (M100‐S24) guidelines for susceptibility testing and EFSA ANS Panel (2012) recommendations for probiotic safety assessment, ensuring a robust evaluation of potential acquired resistance in *B. clausii* and *A. wakoensis*. Antibiotic disks tested included ciprofloxacin (5 μg), ampicillin (10 μg), penicillin G (10 units), chloramphenicol (30 μg), gentamicin (10 μg), cefalexin (30 μg), cephalothin (30 μg), sulfamethoxazole (25 μg), and erythromycin (15 μg). Plates were inoculated with bacterial suspensions adjusted to the 0.5 McFarland standard, incubated at 30°C for 24 h, and inhibition zones measured with a digital caliper (CLSI 2014; EFSA ANS Panel 2012).

### 2.6 Catalase and Hemolytic Tests

Catalase activity in *A. wakoensis* and *B. clausii* (as control) was determined by adding a drop of 5% (v/v) hydrogen peroxide to bacterial colonies on Alkaline *Bacillus* agar. The presence of immediate bubbles around the colonies was considered indicative of positive catalase activity. Hemolysis was assessed using alkaline brain heart infusion agar supplemented with 5% human blood. The 5% human blood used in this study was expired blood obtained from Iran Blood Transfusion Center, Shiraz, Iran, originally collected with informed donor consent for clinical purposes. The use of expired, anonymized blood for in vitro research complies with institutional ethical guidelines and international standards (EDQM 2006; Grachev et al. 1994), which do not require additional consent for such purposes. The plates were then incubated overnight at 30°C. Clear zones surrounding colonies indicated β‐hemolysis, greenish zones α‐hemolysis, and absence of zones γ‐hemolysis (Mohkam, Rasoul‐Amini, et al. 2016; European Committee [Partial Agreement] on Blood Transfusion [CD‐P‐TS] 2017).

All experiments involving human blood utilized expired, anonymized blood from a certified blood transfusion center, collected under donor consent for clinical use. The repurposing of expired blood for in vitro research was conducted in accordance with ethical guidelines from the Ethics Committee of the Shiraz University of Medical Sciences, Shiraz, Iran, and adheres to EDQM (2006).

### 2.7 DNase and Liquefaction of Gelatin

DNase production by *A. wakoensis* and *B. clausii* (as control) along with *Staphylococcus aureus* ATCC 25923 as a positive control and *Escherichia coli* ATCC 25922 as a negative control was evaluated by streaking the strains on DNase agar medium (Merck, Germany). The plates were incubated at 30°C for 48 h. Plates were flooded with 1 N HCl, and the formation of a white opaque background surrounding the transparent colonies was considered positive for DNase activity. Gelatinase production was assessed using tryptone neopeptone dextrose (TND) agar (tryptone 17.0 g, neopeptone 3.0 g, dextrose 2.5 g, NaCl 5.0 g, K_2_HPO_4_ 2.5 g, agar 15 g, in 1000 mL distilled water) containing 0.4% gelatin. Plates were spot‐inoculated with *Bacillus* cells and incubated at 30°C for 2 days. After incubation, the plates were flooded with a saturated ammonium sulfate solution. The appearance of clear zones around colonies against an opaque background was considered positive for gelatinase activity (Mohkam, Rasoul‐Amini, et al. 2016).

### 2.8 Lipase and Lecithinase Activity Assay

Lipase and lecithinase activities were assessed on egg yolk agar composed of Tryptic Soy Agar (Merck, Germany) supplemented with 10% (v/v) sterile egg yolk emulsion and 1% (w/v) NaCl (Sigma‐Aldrich, USA). Plates were incubated at 30°C for 48 h, with turbid zones indicating lipase activity and opaque white zones indicating lecithinase activity (Mohkam, Rasoul‐Amini, et al. 2016).

### 2.9 Protease Assay

Protease activity was assessed on Nutrient Agar (Merck, Germany) supplemented with 2% (w/v) skim milk powder (Merck, Germany) and 1% (w/v) casein powder (Sigma‐Aldrich, USA), both dissolved in distilled water, autoclaved at 121°C for 5 min, and added to autoclaved NA cooled to 45°C–50°C. Plates were spot inoculated with *Bacillus* cultures and incubated at 30°C for 24 h. After incubation, the plates were flooded with 25% trichloroacetic acid (TCA) for 10 min. The appearance of clear zones around the colonies was considered indicative of protease production (Karray et al. 2021).

### 2.10 Fibrinolytic Activity Assay

The bacterial strains were cultivated in a medium containing the following components (per liter): 10 g soya peptone, 10 g yeast extract, 2 g glucose, 1 g MgSO₄, 20 g maltose, and 1000 mL distilled water. The medium was autoclaved at 121°C for 20 min. After cooling, bacterial inoculation was performed, and cultures were incubated at 37°C for 7 days under shaking conditions (150 rpm). Then after, fibrinolytic activity (nattokinase activity) was determined using the fibrin plate method. Fresh‐frozen human plasma containing normal coagulation factors, 300 mg/dL fibrinogen, and the anticoagulant citrate phosphate dextrose adenine (CPDA‐1) was obtained from the blood transfusion center. A portion of the plasma was transferred to a petri dish, and 150 mM calcium chloride (CaCl_2_) at a 9:1 ratio (v/v) was added to recalcify and induce clot formation. After thorough mixing, the plasma was incubated at 37°C for 20 min to allow clot formation. Following coagulation, 10 μL of supernatant from the cultured bacterial strains was added to the center of the petri dish, and the plates were incubated overnight at 37°C. The presence of a clear zone or dissolution of the coagulated plasma around the bacterial supernatant was considered indicative of positive nattokinase enzyme activity (Frias et al. 2021).

### 2.11 Cell Surface Hydrophobicity Test

The cell surface hydrophobicity of the strains was evaluated using the Microbial Adhesion to Hydrocarbon (MATH) assay, following the method by Rosenberg (2006) with slight modifications. Overnight cultures were centrifuged at 8000 rpm for 15 min, and the pellets were washed with phosphate‐buffered saline (PBS, pH 7.2) before being resuspended in the same buffer to achieve an absorbance (*A*_600 nm_) of 1.2–1.6. One milliliter of each bacterial suspension was mixed with an equal volume of ethyl acetate (a monopolar, basic solvent), xylene (an apolar solvent), and chloroform (a monopolar, acidic solvent). The mixture was vortexed for 2 min, incubated at 30°C for 10 min, and vortexed again for an additional 2 min. After standing at room temperature for 20 min, the absorbance of the aqueous phase was measured at 600 nm. The percentage of cell surface hydrophobicity was calculated as follows:

$$\text{Hydophobicity}\;\%=\left(\frac{A_{0}-A}{A_{0}}\right)\times 100$$

where *A*_0_ is the absorbance before extraction and *A* is the absorbance after extraction with organic solvents.

### 2.12 Autoaggregation Assay

The autoaggregation ability of *A. wakoensis* and *B. clausii* (as control) was determined according to the method of Balakrishna (2013) with some modifications. Cultures of *A. wakoensis* and *B. clausii* were grown overnight at 30°C in nutrient broth. The cells were harvested by centrifugation at 8000 rpm for 15 min, washed twice with PBS (pH 7.3), and resuspended in PBS to achieve an absorbance (*A*_600 nm_) of 0.5. Four milliliters of each suspension was vortexed gently for 10 s and incubated at 30°C for 1 h. After incubation, the absorbance of the supernatant was measured at 600 nm. The percentage of autoaggregation was calculated using the following formula:

$$\text{Autoaggregation}\;\%=\left(1-\frac{A_{t}}{A_{0}}\right)\times 100$$

where *A*_0_ is the absorbance at *t* = 0 and *A*_*t*_ is the absorbance at *t* = 1 h.

### 2.13 Biofilm Formation

The ability of bacterial strains to form biofilms was assessed using the microtiter plate method by employing a 96‐well polystyrene microtiter plate, which is commonly used to determine bacterial adhesion to plastic surfaces. Overnight cultures were diluted 1:100 in fresh medium, and 200 μL aliquots (10^6^–10^7^ CFU/mL) were added to 96‐well polystyrene plates. Plates were incubated at 30°C for 24 h. Wells were washed thrice with PBS, stained with 0.1% crystal violet for 15 min, rinsed, and dried. Biofilm biomass was quantified by solubilizing dye with 95% ethanol and measuring absorbance at 570 nm (Coffey and Anderson 2014).

### 2.14 Cytotoxicity Assay

The cytotoxicity assay was performed following the method of Rowan et al. (2001) with modifications. The HepG‐2 cell line was used for this assay. Cell monolayers were seeded at a density of 5 × 10^4^ cells per well in RPMI 1640 medium supplemented with 10% fetal calf serum in 96‐well plates. Cells were then infected with filter‐sterilized (0.2 μm) supernatants from overnight cultures of the test bacteria grown in brain heart infusion (BHI) broth at 30°C. All experiments were repeated in triplicate.

Supernatant samples (100 μL) were added to the cultured cells (in triplicate) immediately after heat treatment at 95°C for 10 min. The monolayers containing the bacterial supernatants were incubated overnight at 37°C in a 5% CO_2_ atmosphere. After overnight incubation, the supernatant from each well was removed, and 25 μL of fresh complete medium (RPMI 1640 + 10% fetal calf serum) containing 0.004 g/mL of MTT reagent (Sigma, Ronkonkoma, NY, USA) was added to the wells. The plates were incubated for 3 h at 37°C in a 5% CO_2_ atmosphere.

The formation of formazan was solubilized by adding 100 μL of dimethyl sulfoxide to each well. Optical densities were measured at 540 nm using an ELISA reader (Biotek, Power Wave, Winooski, VT, USA). Cytotoxicity, expressed as the percentage of dead cells, was calculated using the formula:

$$\text{Cytotoxicity}\;\%=\left(1-\frac{\mathrm{OD}\;\text{of test sample}}{\mathrm{OD}\;\text{of negative control}}\right)\times 100$$

### 2.15 Adhesion and Invasion Studies

The bacterial strains were cultured in BHI broth (Himedia, India) at 30°C for 15–18 h, followed by two washes with PBS. It is important to note that bacteria grown in BHI broth do not form spores. The bacteria were then suspended to a concentration of approximately 10^7^–10^8^ CFU/mL in complete cell culture medium supplemented with HEPES, with optical density readings used to estimate CFU.

Adherence and invasion assays were performed using the HT29 mucus‐secreting cell line, as described by Rowan et al. (2001), with minor modifications. HT29 cells were seeded in 24‐well culture plates at a density of 10^5^ cells per well, and monolayers were grown for a minimum of 20 days at 37°C with 5% CO_2_ in RPMI 1640 medium containing 10% fetal calf serum. Controls included: (1) “bacteria but no cells” wells, incubated with bacterial suspensions (10^−7^ CFU/mL) to determine initial counts (CFU_initial), and (2) uninfected cell‐only wells with HepG‐2 cells in RPMI 1640 with 10% FBS, monitored via microscopy and trypan blue staining (> 95% viability) to confirm monolayer health.

Before the assays, the cell monolayers were washed once with complete culture medium, followed by inoculation with 1 mL of 10^7^–10^8^ CFU of bacteria per well, maintaining a bacteria‐to‐cell ratio of approximately 100:1. The medium was supplemented with 0.01 mol/L HEPES. Incubation was carried out for 2 h at 37°C in a 5% CO_2_ atmosphere, with control wells containing bacteria but no cells incubated in parallel. Following incubation, the monolayers were washed four times with culture medium to remove non‐adherent bacteria. The total number of adherent and invasive bacteria in each well was quantified by lysing the cells with 1 mL of 0.1% Triton X‐100 in water for 5 min at 37°C, followed by plating for viable counts on nutrient agar. The percentage of adherent and invasive bacteria was calculated as follows:

$$\text{Adhesion}\;\%=\left(\text{CFUtotal}/\text{CFUinitial}\right)\times 100$$

$$\text{Invasion}\;\%=\left(\text{CFUinvaded}/\text{CFUinitial}\right)\times 100$$

Invasion assays were conducted concurrently with adhesion assays. After the washing steps, 1 mL of complete culture medium containing 100 μg/mL gentamicin was added to each well, and incubation continued for an additional 2 h at 37°C. As gentamicin kills extracellular bacteria, this allowed for the enumeration of only the bacteria that had invaded the cells. After incubation, the monolayers were washed three times with culture medium to remove the gentamicin, and the invaded bacteria were counted by lysing the cells with 0.1% Triton X‐100 in water (Ramarao and Lereclus 2006). Assays were conducted in triplicate to ensure reproducibility.

### 2.16 Plasmid Extraction

Plasmid extraction was performed using a standard alkaline lysis method. Initially, 1.5 mL of bacterial culture was transferred to a microcentrifuge tube and centrifuged at 13,000 rpm for 1 min to pellet the cells. The supernatant was discarded using aspiration. The bacterial pellet was resuspended in 100 μL of GTE buffer (Glucose‐Tris‐EDTA) along with 10 μL of lysozyme solution. The suspension was mixed thoroughly by pipetting and incubated at 37°C for 30 min to allow enzymatic digestion of the cell wall.

Following incubation, resuspension buffer was added to the solution, and the mixture was gently vortexed or pipetted to ensure complete resuspension. Lysis buffer was then added, and the tube was inverted 3–4 times to mix thoroughly. To neutralize the lysate, neutralization buffer was added, and the solution was rapidly inverted 3–4 times. The lysed cells were then centrifuged at 13,000 rpm for 1 min at 4°C, and the supernatant was carefully transferred to a binding filter column placed in a 2 mL collection tube.

At this stage, 500 μL of denaturation buffer was added to the supernatant in the binding filter column, and the solution was allowed to stand for 5 min to ensure proper binding of plasmid DNA to the column. The column was then centrifuged for 1 min at 13,000 rpm, and the flow‐through was discarded. To wash the DNA, 700 μL of washing buffer was added to the column, followed by centrifugation at 13,000 rpm for 1 min.

The binding filter column was then transferred to a fresh 1.5 mL microcentrifuge tube, and 50 μL of elution buffer was added directly to the filter. The column was incubated for 1 min to allow the elution of plasmid DNA, which was then recovered by centrifugation at 13,000 rpm for 1 min.

To confirm the successful extraction of plasmid DNA, 10 μL of the eluted plasmid DNA was loaded onto a 0.8% agarose gel along with a 1 kbp DNA ladder. Gel electrophoresis was performed at 100 mV, and the plasmid bands were visualized using a gel documentation system (Bigot and Charbit 2009).

### 2.17 Detection of Enterotoxins Using PCR

To screen isolates for the presence of enterotoxin genes (*hblA*, *hblC*, *hblD*, *nheA*, *nheB*, *bceT*, and *cytK*) (Table 1), total DNA was extracted from bacterial cells grown to the log phase (~10^6^ CFU/mL) in BHI broth (Difco) at 37°C. One milliliter of the bacterial culture was centrifuged at 10,000 *g* for 2 min. The resulting pellet was resuspended in 50 μL of sterile water, boiled for 5 min, and centrifuged again at 12,000 *g* for 3 min. The supernatant was transferred to a new microcentrifuge tube and stored at −20°C until use. *B. cereus* PTCC 1047 and *B. cereus* PTCC 1015 were used as positive controls for all above‐mentioned enterotoxin genes.

**TABLE 1:** Specific toxin primers used in this study.

Target gene	Primer's name	Primer sequence (5′ → 3′)	Amplicon size (bp)	References
*hblA*	hblA for	GCAAAATCTATGAATGCCTA	1025	Ngamwongsatit et al. (2008)
	hblA rev	GCATCTGTTCGTAATGTTTT		
*hblC*	hblC for	CCTATCAATACTCTCGCAA	351	Ngamwongsatit et al. (2008)
	hblC rev	TTTCCTTTGTTATACGCTGC		
*hblD*	hblD for	GAA ACAGGG TCTCATATTCT	1018	Ngamwongsatit et al. (2008)
	hblD rev	CTGCATCTTTATGAATATCA		
*nheA*	nheA for	ATTACAGGGTTATTGGTTACGCAGT	625	Yang et al. (2005)
	nheA rev	AATCTTGCTCCATACTCTCTTGGATGCT		
*nheB*	nheB for	GTGCAGCAGCTGTAGGCGGT	325	Yang et al. (2005)
	nheB rev	ATGTTTTTCCAGCTATCT TTCGCAAT		
*bceT*	bceT for	GACTACATTCACGATTACGCAGAA	303	Lee et al. (2008)
	bceT rev	CTATGCTGACGAGCTACATCCATA		
*cytK*	cytK for	GGCGCTAGTGCAACA TTACG	800	Yang et al. (2005)
	cytK rev	TCATACCAGGAGAGAAACCGC		

PCR amplification was carried out using a Gene Cycler (Bio‐Rad). Each PCR reaction contained 20 pmol of each primer (Tables 3 and 4), 10 μL of PCR 2× Taq premix (Pars Tous, Iran), and 1 μL (100 ng) of template DNA in a final volume of 20 μL. The PCR conditions included an initial denaturation at 94°C, followed by 30 cycles of 94°C for 30 s, 54°C for 1 min, and 72°C for 2 min, with a final extension at 72°C for 7 min. PCR products were analyzed on a 1% (w/v) agarose gel (Cinnagen, Tehran, Iran) and visualized under UV light.

### 2.18 Enterotoxin Detection by Immunoassay Kits

Enterotoxins were detected using two commercial immunoassay kits. The HblC subunit of the Hbl enterotoxin was detected in enrichment cultures using the BCET‐RPLA kit (Oxoid). The NheA subunit of the Nhe enterotoxin was identified using the Tecra BDE kit (Tecra Diagnostics).

### 2.19 Statistical Analysis

All experiments were performed in triplicate. Averages and standard errors were calculated using GraphPad Prism software (version 9.0).

## 3 Results

### 3.1 Screening of Probiotic Features

#### 3.1.1 Acid and Bile Salts Tolerance

The survival rates of *A. wakoensis* were assessed after 3 h of exposure to acidic conditions at pH 2.5. As presented in Table 2, *S. clausii* (as control) demonstrated a survival rate of 96.7%, indicating a high level of resilience in acidic environments similar to those found in the stomach. In contrast, *A. wakoensis* showed a slightly lower survival rate of 95.6%, which still reflects considerable acid tolerance. Additionally, the resistance of these strains to bile salts was evaluated using a 0.5% oxgall media over a 24‐h incubation period. Bile salt tolerance was qualitatively assessed, with *A. wakoensis* and *S. clausii* exhibiting visible colony growth on alkaline TSA with 0.5% oxgall, comparable to oxgall‐free controls, indicating robust tolerance.

**TABLE 2:** Survival rates of *A. wakoensis* after 3 h of exposure to acidic conditions (pH 2.5).

Bacterial strains	Initial count (log), *t* = 0 h.	Final count (log), *t* = 3 h.	Survival rate (%)
*S. clausii*	6.041 ± 0.05	5.845 ± 0.07	96.75 ± 1.2
*A. wakoensis*	6.642 ± 0.08	6.176 ± 0.06	95.57 ± 0.9

*Note:* Values represent viable bacterial counts in log CFU/mL.

#### 3.1.2 Determination of Antimicrobial Activity

The antimicrobial activities of *A. wakoensis* and *S*. *clausii* (control) were assessed against eight bacterial strains, and the minimum inhibitory concentration (MIC) and minimum bactericidal concentration (MBC) were determined. The results are presented in Table 3. *A. wakoensis* also exhibited antimicrobial activity against all tested strains. The MIC values ranged from 1/32 to 1/2, with the lowest MIC (1/32) again observed against *B. cereus* . The MBC values for *A. wakoensis* ranged from 1/16 to 1/2. *S. clausii* demonstrated inhibitory activity against all tested strains, with MIC values ranging from 1/64 to 1/4. The lowest MIC (1/64) was observed against *B. cereus* , indicating the highest sensitivity. MBC values for *S. clausii* ranged from 1/32 to 1/8. Notably, an MBC was not determined for *P. aeruginosa* . In comparison to the control ( *S. clausii* ), *A. wakoensis* showed comparable to slightly reduced inhibitory and bactericidal activities. *P. aeruginosa* displayed the lowest susceptibility to both *S. clausii* and *A. wakoensis*.

**TABLE 3:** The minimum inhibitory concentration (MIC) and minimum bactericidal concentration (MBC) of *B. clausii* and *A. wakoensis* against different bacterial strains.

Bacterial strain	*S. clausii*		*A. wakoensis*	
	MIC	MBC	MIC	MBC
*S. aureus* ATCC 25923	1/16	1/8	1/8	1/4
*S. epidermidis* ATCC 12228	1/32	1/16	1/16	1/8
*P. aeruginosa* ATCC 27853	1/4	—	1/2	1/2
*B. cereus* ATCC 11778	1/64	1/32	1/32	1/16
*P. vulgaris* ATCC 13315	1/8	1/4	1/4	1/2
*E. coli* ATCC 25922	1/16	1/8	1/8	1/4
*S. typhi* ATCC 6539	1/32	1/16	1/16	1/8
*S. dysenteriae* ATCC 11835	1/32	1/16	1/16	1/8

#### 3.1.3 Catalase, Protease, Nattokinase and Hemolytic Tests

*A. wakoensis* showed catalase activity, as indicated by bubble formation when 5% (v/v) hydrogen peroxide was applied to the colonies. This reaction confirms the presence of the catalase enzyme, which breaks down hydrogen peroxide into water and oxygen. This bacterium also exhibited protease production on skimmed milk agar, as evidenced by clear zones around the colonies. Additionally, nattokinase activity was observed, indicated by the dissolution of the coagulated plasma around the bacterial supernatant. For hemolytic activity, the bacterium was streaked onto blood agar plates containing 5% human blood and incubated at 37°C for 24 h. No clear zones of hemolysis were observed, indicating that neither strain exhibited β‐hemolytic activity, classifying them as non‐hemolytic.

#### 3.1.4 Lipase, Lecithinase, DNase Assay, Liquefaction of Gelatin and Plasmid Extraction

The ability of *A. wakoensis* to produce DNase was tested on DNase agar plates. After 48 h of incubation at 30°C, no clear zones around the colonies were observed, indicating that neither strain produces the DNase enzyme. Similar results were observed for lipase and lecithinase production, with both tests yielding negative results. Additionally, gelatin liquefaction was assessed by spotting the strains onto TND agar plates containing 0.4% gelatin. The plates were incubated at 30°C for 3 days and then flooded with a saturated ammonium sulfate solution. Fairly clear zones around the colonies were observed, indicating that both *A. wakoensis* and *B. clausii* (as control) were capable of gelatin liquefaction, indicating the faint production of gelatinase enzymes. Finally, the plasmid extraction results showed that both bacterial strains lacked plasmids.

#### 3.1.5 Antibiotic Susceptibility Tests

The antibiotic susceptibility of *A. wakoensis* was evaluated by measuring the diameter of the inhibition zones (in mm) around bacterial colonies after exposure to various antibiotics (Table 4). *A. wakoensis* showed the largest inhibition zone in response to chloramphenicol (30 mm), followed by gentamicin (21 mm), penicillin (18 mm), cephalothin (17 mm), and ciprofloxacin (15 mm). Moderate inhibition zones were observed for cephalexin (12 mm) and tetracycline (10 mm). No inhibition zones were detected for cefixime, novobiocin, nalidixic acid, erythromycin, or sulphamethoxazole, indicating resistance to these antibiotics. On the other hand, *B. clausii* served as a control, displayed the largest inhibition zone for sulphamethoxazole (24 mm) and chloramphenicol (38 mm). Inhibition zones were also observed for erythromycin (17 mm), cephalothin (16 mm), penicillin (15 mm), cephalexin (14 mm), and gentamicin (18 mm). For tetracycline, *A. wakoensis* exhibited a larger zone of inhibition compared to *B. clausii* , which showed a smaller zone of inhibition measuring 8 mm. Additionally, no inhibition zones were observed for cefixime, novobiocin, nalidixic acid, or ciprofloxacin, indicating resistance in both strains.

**TABLE 4:** Antibiotic susceptibility pattern of *A. wakoensis* and *B. clausii* .

The diameter of the lack of growth halo (mm)		
Antibiotic	*A. wakoensis*	*B. clausii*
Cloramphenicol	30	38
Cefixime	6	6
Tetracycline	10	8
Novobiocin	6	6
Ciprofloxacin	15	6
Nalidixic acid	6	6
Penicillin	18	15
Gentamicin	21	18
Erythromycin	6	17
Cephalexin	12	14
Cephalothin	17	16
Sulphamethoxazol	0	24

#### 3.1.6 Auto‐Aggregation and Cell Surface Hydrophobicity Assay

The cell surface hydrophobicity of *A. wakoensis* was evaluated using three different solvents: xylene (apolar), ethyl acetate (monopolar, basic), and chloroform (monopolar, acidic), as shown in Table 5.

**TABLE 5:** The percentage of bacteria's affinity for various solvents.

Solvent	The percentage of bacteria's affinity for solvent	
	*A. wakoensis*	*B. clausii*
Xylene	47.09 ± 3.12	12.125 ± 1.45
Ethyl acetate	17.56 ± 1.30	8.06 ± 0.45
Chloroform	39.18 ± 4.11	21.05 ± 2.39

*A. wakoensis* exhibited the highest affinity for xylene, with a hydrophobicity of 47.09%, indicating a strong interaction with this apolar solvent. The affinity for chloroform was 39.18%, while the lowest affinity was observed with ethyl acetate, which had a hydrophobicity of 17.56%. In contrast, the control showed significantly lower hydrophobicity in all solvents compared to *A. wakoensis*. The highest affinity for the control was observed for chloroform (21.05%), followed by xylene (12.12%) and ethyl acetate (8.06%).

In the auto‐aggregation assay, *A. wakoensis* displayed an auto‐aggregation percentage of 7.01% ± 1.02%, reflecting its ability for cell‐to‐cell aggregation, while the control bacterium ( *B. clausii* ) exhibited a slightly lower auto‐aggregation percentage of 6.0% ± 0.98%.

#### 3.1.7 Biofilm Formation Assay

The biofilm‐forming abilities of *A. wakoensis* were assessed by measuring the optical density (OD) at 570 nm while *B. clausii* (as control) exhibited a higher optical density (OD 570 nm) of 1.118, suggesting a stronger biofilm formation compared to *A. wakoensis*, which showed a lower optical density of 0.74.

#### 3.1.8 Adhesion and Invasion Assay

The ability of *A. wakoensis* to adhere to and invade HT‐29 cells was assessed after 18 h of growth in BHI broth. The results showed species‐specific differences in adhesion and invasion capacities (Table 6). The *A. wakoensis* demonstrated adhesion to HT‐29 cells, with a relatively high adhesive capacity. In contrast, this bacterium showed a relatively weak ability to invade HT‐29 cells, which is comparable to *B. clausii* .

**TABLE 6:** Adhesion and invasion assay of seven *Bacillus* isolates.

*Bacillus* strain	Adhesion (%)	Invasion (%)
*A. wakoensis*	0.11	0.01
*B. clausii*	0.5	0.01

#### 3.1.9 Cytotoxicity Assay

**FIGURE 1:**
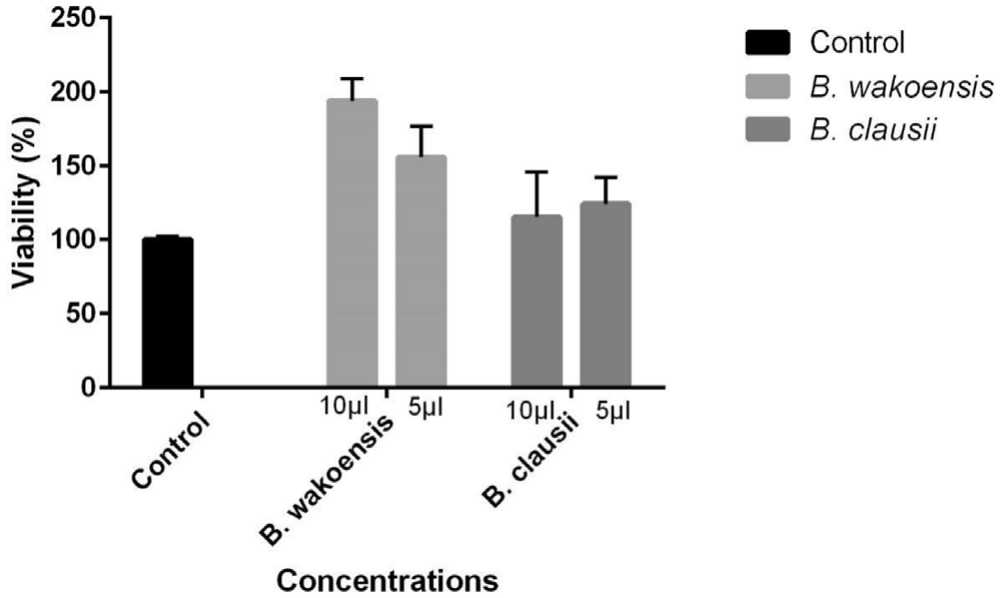
Cytotoxicity assay showing the percentage of cell viability after treatment with 10 and 5 μL concentrations of *A. wakoensis* and *S. clausii* supernatants. Control represents untreated cells. Error bars indicate standard deviations. Data represent means ± SD from three replicates. One‐way ANOVA with Dunnett's test showed significant differences from control (*p* < 0.05).

The cytotoxicity of *A. wakoensis* was evaluated using different concentrations of bacterial supernatants (10 and 5 μL), with untreated cells serving as the control (Figure 1). At the 10 μL concentration, *A. wakoensis* exhibited the highest cell viability, reaching approximately 200% compared to the control. Although viability decreased slightly at the 5 μL concentration, it remained high at around 150%. These findings suggest that *A. wakoensis* significantly enhances cell viability at both concentrations tested, demonstrating its potential beneficial effects on cell health.

#### 3.1.10 Enterotoxin Detection

The enterotoxin detection assay was conducted to determine the presence of specific enterotoxin genes in *A. wakoensis*. PCR analysis targeted the genes *hblA*, *hblC*, *hblD*, *nheA*, *nheB*, *bceT*, and *cytK*, which revealed that all enterotoxin genes were absent.

For confirmation of the absence of enterotoxins, further investigation was performed using commercial *Bacillus* enterotoxin detection kits, including the BCET‐RPLA kit and the Tecra BDE kit. Both *A. wakoensis* and *B. clausii* (as control) tested negative for the presence of enterotoxins with the BCET‐RPLA and Tecra BDE kits, supporting the PCR findings.

## 4 Discussion

The probiotic potential of *A. wakoensis* was evaluated through various in vitro assays examining their ability to tolerate acid and bile, antimicrobial effects, adhesion to epithelial cells, antibiotic susceptibility, and enzyme production. This study is based on existing research works on *Bacillus* species as probiotics, highlighting their resilience under harsh conditions and potential role in promoting gastrointestinal health. The choice of *A. wakoensis* is particularly intriguing, as this alkaliphilic species is primarily known for its enzyme production capabilities and ability to survive in extreme pH environments (Danilova and Sharipova 2020; Romero et al. 2020). Traditionally viewed as an industrial bacterium, our exploration of its probiotic potential aligns with recent interests in using alkaliphilic bacteria for health applications. As a well‐documented probiotic, *B. clausii* ATCC 700160 served as a valuable control strain, allowing comparison of its established benefits with the newly studied *A. wakoensis*. Both strains exhibited robust resistance to environmental challenges, suggesting their potential utility as probiotics suited to the human gut, where factors such as acidity and bile salts often limit bacterial survival (Ghelardi et al. 2015; Sadrimovahed and Ulusoy 2024).

A fundamental requirement for effective probiotics is the ability to withstand the acidic environment of the stomach and the bile salts in the small intestine (Tsifintaris et al. 2024; Vasiee et al. 2022; Kardooni et al. 2023b; Falah et al. 2021). In this study, *A. wakoensis* demonstrated high acid tolerance, with survival rates of 95.57% after 3 h at pH 2.5. These findings are consistent with prior research on *Bacillus* strains, with studies such as Werdi and Al‐Hadidy (2023) Chelliah et al. (2024) showing *B. clausii* 's significant survival under similar acidic conditions. The bile salt tolerance test further demonstrated that both strains could survive in the presence of 0.5% oxgall, underscoring their resilience in gastrointestinal environments. This aligns with research on other *Bacillus* probiotics like *B. coagulans* (Ganeden bc30), which exhibit similar robustness, further positioning *Bacillus* species as suitable candidates for probiotic applications due to their ability to endure gastrointestinal stressors (Nyangale et al. 2014). Unlike *Lactobacillus* and *Bifidobacterium*, which are often sensitive to such conditions, *Bacillus* spp. offer a more resilient option for probiotic formulations (Celandroni et al. 2019).

A key factor in promoting gut health is the antimicrobial activity of probiotic bacteria, which helps prevent infections by competing with pathogenic microorganisms. In this study, the antimicrobial activity of *A. wakoensis* was compared to that of *B. clausii* (control) against a panel of bacterial strains by determining their minimum inhibitory concentrations (MICs) and minimum bactericidal concentrations (MBCs). *A. wakoensis* exhibited antimicrobial activity against all tested strains, demonstrating its potential as a source of antimicrobial compounds. The MIC values ranged from 1/32 to 1/2, with the lowest MIC (1/32) observed against *B. cereus* . The MBC values for *A. wakoensis* ranged from 1/16 to 1/2. When compared to the control ( *B. clausii* ), *A. wakoensis* generally exhibited comparable to slightly reduced inhibitory and bactericidal activities. *B. clausii* demonstrated MIC values ranging from 1/64 to 1/4, with a lowest MIC of 1/64 against *B. cereus* , and MBC values ranging from 1/32 to 1/8. While *B. clausii* has been extensively studied for its antimicrobial properties (Jeon et al. 2017; Dhakephalkar et al. 2024; Fateminasab et al. 2023), it is important to note that the antimicrobial spectrum of *Bacillus* species, including *B. clausii* , can exhibit significant strain‐to‐strain variability and can also be influenced by the specific methodologies and concentrations used in antimicrobial assays. While some reports might indicate limited efficacy of certain *B. clausii* strains against Gram‐negative bacteria (Urdaci et al. 2004), other studies do provide evidence of *B. clausii* exerting inhibitory effects against Gram‐negative pathogens. For instance, studies have highlighted that certain *B. clausii* strains can produce bacteriocin‐like inhibitory substances or other antimicrobial compounds that are effective against a range of bacteria, including Gram‐negatives like *E. coli* and *Salmonella* species (Rochín‐Medina et al. 2018; Devi et al. 2008). This supports the findings in our study where the control *B. clausii* strain demonstrated activity against the tested Gram‐negative organisms. Such activity underscores the potential for specific *B. clausii* strains to contribute to the modulation of gut microbiota by inhibiting not only Gram‐positive but also potentially pathogenic Gram‐negative bacteria. On the other hand, *A. wakoensis* is less well‐characterized. In this regard, the fact that both species exhibited the highest activity against *B. cereus* suggests potential similarities in their mechanisms of action or target sites within this bacterium. Further investigation is needed to elucidate the specific compounds responsible for the antimicrobial activity of *A. wakoensis* and to determine whether they are related to those produced by *B. clausii* or other *Bacillus* species.

The observed MIC and MBC ranges for *A. wakoensis* are within the range reported for other *Bacillus* species (Fateminasab et al. 2023; Golnari et al. 2024; Fayed et al. 2023). However, variations in antimicrobial activity are common among different *Bacillus* species and strains, reflecting differences in their genetic makeup, metabolic pathways, and the specific antimicrobial compounds they produce (Golnari et al. 2024; Tran et al. 2022). Notably, *P. aeruginosa* displayed the lowest susceptibility to *A. wakoensis*, with the highest MIC and MBC values. This aligns with the known intrinsic resistance of *P. aeruginosa* to many antimicrobials (Langendonk et al. 2021; Fernández‐Billón et al. 2023). Future research should focus on identifying and characterizing the specific mechanisms by which *A. wakoensis* exerts its antimicrobial effects, particularly against recalcitrant organisms like *P. aeruginosa* . While our CFS‐based assays provide preliminary evidence of extracellular antimicrobial compounds, potentially including bacteriocins, they do not constitute specific detection (e.g., via protease sensitivity or genetic screening). Bacteriocin production is a desirable probiotic trait for pathogen inhibition but is not a sole reliable index; it must be evaluated alongside acid/bile tolerance, adhesion, safety, and in vivo efficacy in accordance with FAO/WHO (2002) guidelines.

*A. wakoensis* demonstrated significant enzymatic activity, which enhances its probiotic potential by contributing to gut health. This strain displayed catalase and protease activity, suggesting its ability to neutralize oxidative stress and support protein digestion, respectively. This activity aligns with other probiotic *Bacillus* strains, such as *B. subtilis* and *B. coagulans* , which are also known for their catalase production and resilience against oxidative damage (Adibpour et al. 2019). Protease production, in particular, facilitates protein digestion, a benefit that may enhance nutrient absorption in the host's gastrointestinal tract. The faint gelatinase activity observed in both strains suggests minimal production of gelatinase, which is often associated with pathogenicity. However, the weak expression observed here contrasts with pathogenic strains where high gelatinase activity contributes to tissue damage. This minimal activity is consistent with other probiotic *Bacillus* strains, which either lack gelatinase or express it at levels too low to be harmful (Jiang et al. 2023).

Interestingly, *A. wakoensis* exhibited nattokinase activity, which has cardiovascular benefits, such as reducing blood clot formation. Although nattokinase is not directly related to gut health, its presence in *A. wakoensis* adds an extra functional benefit that may expand its potential applications to cardiovascular health. Strains like *B. subtilis natto* are commercially popular due to nattokinase production, supporting their safety profile for functional food applications (Sadrimovahed and Ulusoy 2024; Raphel and Halami 2024; Mohkam, Nezafat, et al. 2016). The effectiveness of nattokinase in cardiovascular health is significantly enhanced by its ability to transfer through the gut and enter the bloodstream. Research indicates that nattokinase can be absorbed from the gastrointestinal tract, as demonstrated in an in vivo study where intact absorption of the enzyme was observed in rats, leading to prolonged degradation of plasma fibrinogen. This absorption is crucial for its fibrinolytic activity, which helps in dissolving blood clots and improving blood flow (Fujita et al. 1995, 2011). The presence of nattokinase in both *A. wakoensis* and *B. clausii* could enhance the commercial viability of these strains as multifunctional probiotics, offering not only gut health benefits but also cardiovascular advantages. This dual functionality makes them attractive candidates for applications in both digestive and cardiovascular health supplements.

The adhesion of probiotics to intestinal epithelial cells is essential for colonization and persistence in the gut. In this study, *A. wakoensis* demonstrated measurable adhesion to HT‐29 cells, with *S. clausii* (as control) showing a slightly higher adhesion rate than *A. wakoensis*. These results align with previous studies on *S. clausii* , which is known for its effective adhesion to intestinal epithelial surfaces (Dhakephalkar et al. 2024; Gumus et al. 2024). Although adhesion for both strains is generally lower than that of some colonizing *Lactobacillus* strains, *A. wakoensis*'s adhesion suggests its potential as a probiotic, particularly given its industrial applications and alkaliphilic characteristics. For spore‐forming *Bacillus* species, even modest adherence is functionally relevant, facilitating mucus layer interactions or competitive exclusion of pathogens by occupying binding sites on the intestinal mucosa, a trait also observed in other *Bacillus* probiotics like *B. subtilis* and *B. coagulans* (Sui et al. 2020). The minimal invasion rate (< 0.01%) for both strains underscores their safety, minimizing risks of translocation and confirming their non‐pathogenic nature, a common feature among *Bacillus* probiotics that balances adhesion with minimal invasion (Mohkam, Rasoul‐Amini, et al. 2016). In this regard, the HT‐29 cell line provides specific insights into interactions with the colonic mucosa, and future studies using Caco‐2 cells, as a model for the small intestine's absorptive surface, will enable comparative analysis across different gut epithelial environments.

Safety concerns about probiotics often focus on antibiotic susceptibility and the potential for plasmid‐mediated gene transfer. This study found that *A. wakoensis* exhibited species‐specific variations in antibiotic susceptibility. In this context, *A. wakoensis* showed high sensitivity to chloramphenicol, gentamicin, and penicillin, suggesting it would not contribute to resistance in clinical settings. This strain also exhibited resistance to certain antibiotics, a trait common among *Bacillus* species but generally intrinsic and non‐transferable. This characteristic was also observed in other *Bacillus* probiotics, such as *B. subtilis* and *B. coagulans* , which often show resistance to erythromycin and tetracycline without evidence of gene transfer (Haque et al. 2024). The absence of plasmids in both strains further strengthens their safety profile, as plasmids can often harbor virulence genes and facilitate gene transfer in the gut microbiome (Bang et al. 2021). This absence reduces risks associated with horizontal gene transfer, making *B. wakoensis* and *B. clausii* safer for probiotic applications. In this context, antibiotic resistance in probiotic strains is a double‐edged sword because intrinsic and chromosomally encoded resistance can help ensure the persistence of a probiotic during antibiotic treatment, whereas the presence of mobile and transferable elements can pose a safety risk through potential horizontal gene transfer to pathogenic gut bacteria. According to EFSA ANS Panel (2012) and FAO/WHO (2002) recommendations, probiotics intended for human use should not carry resistance genes on mobile genetic elements such as plasmids or transposons, and any resistance should ideally be intrinsic and non‐transferable. In the present study, *A. wakoensis* showed resistance to some antibiotics but no plasmids were detected, and its resistance pattern aligns with previous reports on *Bacillus* species where resistance traits, particularly to β‐lactams and aminoglycosides, are often non‐transferable. Together, the absence of plasmids and enterotoxin genes supports the classification of *A. wakoensis* as a safe candidate in line with EFSA and FAO guidelines. This intrinsic, non‐transferable resistance profile may also provide a functional advantage by allowing the strain to remain active during antibiotic therapy, thereby maintaining its probiotic functions without elevating the risk of spreading antimicrobial resistance genes. This balance between safety and persistence highlights the potential of *A. wakoensis* in probiotic applications and provides a foundation for exploring its use in conditions where antibiotic treatment is unavoidable.

Biofilm formation, autoaggregation, and cell surface hydrophobicity are essential traits that support probiotic adhesion and colonization of the gut (Mohkam, Rasoul‐Amini, et al. 2016; Rouhi et al. 2024; Behbahani et al. 2024; Kardooni et al. 2023a). *B. clausii* (as control) demonstrated stronger biofilm formation than *B. wakoensis* , consistent with its clinical effectiveness in gastrointestinal applications. Biofilm formation enhances a probiotic's ability to adhere to surfaces, which is vital for persistence in the gut (Mohkam, Rasoul‐Amini, et al. 2016; Celandroni et al. 2019). Although *A. wakoensis* exhibited lower biofilm formation, its ability to survive in alkaline environments indicates potential for future probiotic applications. Autoaggregation, the ability of bacterial cells to adhere to one another, also contributes to forming stable microbial communities that prevent pathogen colonization (Mohkam, Rasoul‐Amini, et al. 2016; Celandroni et al. 2019). This strain demonstrated moderate autoaggregation, which is in line with other *Bacillus* probiotics and suggests their potential to establish stable gut populations (Balakrishna 2013; Chelliah et al. 2024; Dhakephalkar et al. 2024). Additionally, *A. wakoensis* showed higher hydrophobicity than *B. clausii* , particularly with xylene and chloroform, suggesting strong interactions with the intestinal mucosa. This high hydrophobicity may enhance *A. wakoensis*'s adhesion to epithelial cells, a trait often associated with effective colonization (Mohkam, Rasoul‐Amini, et al. 2016; Ritter et al. 2018).

To ensure the therapeutic potential and safety of probiotics, it is essential that probiotic bacteria demonstrate non‐cytotoxicity, support host cell viability, and lack active toxin production (Elshaghabee et al. 2017; Chelliah et al. 2024; Ritter et al. 2018; Cutting 2011; Barzegar et al. 2021). The observed increase in HepG2 cell viability, reaching nearly 200% of *A. wakoensis* supernatant, suggests that the bacterial supernatants contain bioactive compounds that promote cell growth or survival. Bacterial supernatants, particularly from *Bacillus* species, are known to harbor a variety of metabolites, including growth factors, vitamins, amino acids, and other bioactive molecules that can influence eukaryotic cell behavior (Ozone et al. 2002; Seo et al. 2000). For instance, *B. subtilis* has been shown to produce cyclic dipeptides, such as cyclo‐(Val‐Leu) and cyclo‐(Val‐Ile), which act as bifidogenic growth factors, stimulating the proliferation of *Bifidobacterium* species (Hatanaka et al. 2020). While these studies focus on bacterial interactions, they highlight the potential of *Bacillus* supernatants to contain compounds that can modulate cell growth. Similarly, *B. mesentericus* has been reported to secrete a growth‐promoting factor, 3,3‐dihydroxyazetidine, which enhances the growth of *Bifidobacterium* (Halloran and Underwood 2019). These findings suggest that *A. wakoensis* and *S. clausii* may produce analogous compounds that support the viability of HepG2 cells. Additionally, bacterial supernatants can exert protective effects on eukaryotic cells by modulating inflammatory pathways or providing antioxidant properties (Plaza‐Diaz et al. 2019). For example, supernatants from probiotic bacteria like *Lactobacillus* and *Bifidobacterium* have been shown to reduce pro‐inflammatory cytokines and promote cell survival in various cell types (Pellegrini et al. 2020). Although direct studies on *A. wakoensis* or *S. clausii* with HepG2 cells are limited, the general properties of *Bacillus* supernatants indicate that they may contain nutrients, growth factors, or anti‐inflammatory agents that contribute to the observed increase in HepG2 cell viability. In this context, the findings affirm that *A. wakoensis* not only meets but exceeds essential safety benchmarks, demonstrating both non‐cytotoxicity and the ability to support host cell viability. However, further characterization of the supernatant components could identify specific bioactive molecules responsible for this effect, providing deeper insights into the mechanisms of bacterial‐eukaryotic cell interactions. From a safety perspective, both *A. wakoensis* and the reference strain *B. clausii* passed several key assessments recommended for preliminary probiotic evaluation. Neither strain demonstrated β‐hemolytic activity, DNase production, lipase or lecithinase activity, nor plasmid carriage. PCR screening and immunoassays confirmed the absence of major *Bacillus* enterotoxin genes (*hblA*, *hblC*, *hblD*, *nheA*, *nheB*, *bceT*, and *cytK*) and their corresponding protein products. Antibiotic susceptibility profiling revealed sensitivity to multiple clinically relevant antibiotics, suggesting a low risk of harboring transmissible resistance genes. Additionally, cytotoxicity testing via MTT assay indicated no adverse effects on mammalian epithelial cell viability. While these results support a favorable preliminary safety profile, further investigations would be necessary before considering *A. wakoensis* for human consumption. Such evaluations should include whole‐genome sequencing to detect any cryptic virulence or resistance determinants, in vivo safety and toxicity testing in appropriate animal models, assessment of D‐lactate and biogenic amine production, and controlled human intervention trials to confirm safety and tolerability in target populations.

While the in vitro assays in this study provide valuable preliminary insights into the probiotic potential of *A. wakoensis*, it is important to acknowledge the limitations of this approach. The results, derived from controlled laboratory conditions, may not fully replicate the complex interactions within the human gastrointestinal tract. Therefore, in vivo studies are essential to confirm the efficacy and safety of *A. wakoensis* in a living system. Additionally, whole‐genome sequencing is necessary to provide a comprehensive assessment of the strain's genetic makeup, ensuring the absence of undetected virulence factors or resistance genes.

To further establish *A. wakoensis* as a viable probiotic candidate, future research should focus on in vivo studies to validate its safety, colonization potential, and therapeutic effects in living systems. Animal trials will be essential to assess the strain's ability to survive and function within the gastrointestinal tract, as well as its influence on gut microbiota diversity and immune responses, particularly in disease‐specific models. Concurrently, whole‐genome sequencing will provide a comprehensive assessment of the strain's genetic makeup, confirming the absence of virulence factors or resistance genes while identifying unique probiotic traits. This genomic insight will not only reinforce the safety profile of *A. wakoensis* but also enable comparative analyses with established probiotics, potentially uncovering novel functional attributes.

In addition to in vivo and genomic studies, mechanistic investigations are warranted to elucidate the molecular underpinnings of *A. wakoensis*'s probiotic properties, such as its antimicrobial activity and interactions with host cells. Understanding these mechanisms will inform targeted applications and optimize its therapeutic potential. Furthermore, formulation development will be crucial to ensure the strain's viability and stability across diverse storage conditions and gastrointestinal environments, enhancing its efficacy for practical use. Ultimately, clinical trials in human subjects will be necessary to confirm the safety, tolerability, and efficacy of *A. wakoensis*, particularly in populations with gut dysbiosis or specific health conditions. Comparative analyses with other probiotics and exploration of synergistic effects with prebiotics or microbial consortia will also provide valuable insights into its competitive advantages and potential for integration into multi‐strain formulations. These efforts will bridge existing knowledge gaps, providing a robust foundation for the development and potential commercialization of A. wakoensis as a next‐generation probiotic.

## 5 Conclusion

This study provides an initial in vitro characterization of *Alkalihalobacillus wakoensis* PTCC 1596 as a potential probiotic candidate, benchmarked against *Bacillus clausii* ATCC 700160. Both strains demonstrated key probiotic features, including resilience to simulated gastrointestinal conditions (acid pH 2.5, 0.5% bile salts) and production of potentially beneficial enzymes like catalase and protease. *A. wakoensis* exhibited notable cell surface hydrophobicity and qualitative nattokinase activity, suggesting unique attributes. Crucially, both strains showed favorable safety profiles: non‐hemolytic, non‐cytotoxic on HepG‐2 cells, lacking detectable plasmids and screened enterotoxin genes, and possessing manageable antibiotic susceptibility patterns. While *B. clausii* showed stronger biofilm formation, the overall profile of *A. wakoensis* supports its potential for further development. These findings highlight *A. wakoensis* as a promising candidate, particularly given its alkaliphilic nature, though further comprehensive in vivo and genomic safety assessments are necessary to fully establish its efficacy and safety for probiotic applications.

## Conflicts of Interest

The authors declare no conflicts of interest.

## Data Availability

The authors have nothing to report.
